# Ritonavir blocks AKT signaling, activates apoptosis and inhibits migration and invasion in ovarian cancer cells

**DOI:** 10.1186/1476-4598-8-26

**Published:** 2009-04-22

**Authors:** Sanjeev Kumar, Christopher S Bryant, Sreedhar Chamala, Aamer Qazi, Shelly Seward, Jagannath Pal, Christopher P Steffes, Donald W Weaver, Robert Morris, John M Malone, Masood A Shammas, Madhu Prasad, Ramesh B Batchu

**Affiliations:** 1 Laboratory of Surgical Oncology & Developmental Therapeutics, Department of Surgery, Wayne State University, Detroit, MI, USA; 2 Dept of Ob/Gyn, Wayne State University, Detroit, MI, USA; 3 Karmanos Cancer Institute, Detroit, MI 48201, USA

## Abstract

**Background:**

Ovarian cancer is the leading cause of mortality from gynecological malignancies, often undetectable in early stages. The difficulty of detecting the disease in its early stages and the propensity of ovarian cancer cells to develop resistance to known chemotherapeutic treatments dramatically decreases the 5-year survival rate. Chemotherapy with paclitaxel after surgery increases median survival only by 2 to 3 years in stage IV disease highlights the need for more effective drugs. The human immunodeficiency virus (HIV) infection is characterized by increased risk of several solid tumors due to its inherent nature of weakening of immune system. Recent observations point to a lower incidence of some cancers in patients treated with protease inhibitor (PI) cocktail treatment known as HAART (Highly Active Anti-Retroviral Therapy).

**Results:**

Here we show that ritonavir, a HIV protease inhibitor effectively induced cell cycle arrest and apoptosis in ovarian cell lines MDH-2774 and SKOV-3 in a dose dependent manner. Over a 3 day period with 20 μM ritonavir resulted in the cell death of over 60% for MDAH-2774 compared with 55% in case of SKOV-3 cell line. Ritonavir caused G1 cell cycle arrest of the ovarian cancer cells, mediated by down modulating levels of RB phosphorylation and depleting the G1 cyclins, cyclin-dependent kinase and increasing their inhibitors as determined by gene profile analysis. Interestingly, the treatment of ritonavir decreased the amount of phosphorylated AKT in a dose-dependent manner. Furthermore, inhibition of AKT by specific siRNA synergistically increased the efficacy of the ritonavir-induced apoptosis. These results indicate that the addition of the AKT inhibitor may increase the therapeutic efficacy of ritonavir.

**Conclusion:**

Our results demonstrate a potential use of ritonavir for ovarian cancer with additive effects in conjunction with conventional chemotherapeutic regimens. Since ritonavir is clinically approved for human use for HIV, drug repositioning for ovarian cancer could accelerate the process of traditional drug development. This would reduce risks, limit the costs and decrease the time needed to bring the drug from bench to bedside.

## Background

Ovarian cancer is the second most common gynecologic malignancy, but the most common cause of death among women who develop gynecologic cancers [1]. It is the fifth leading cause of cancer death in females in the United States. It is estimated that 22,430 new cases along with 15,280 deaths were attributed to ovarian cancer in 2007 in the United States [1]. Although current management strategies have resulted in a several fold increase in the median survival for ovarian cancer over past few decades, mortality from the disease still remains high [2]. Up to one third of the patients who receive the first line platinum based chemotherapy for ovarian cancer fail to achieve clinical remission and approximately 50% patients who achieve clinical remission in first course of chemotherapy, eventually have relapse of their disease[2]. Both of the above mentioned categories of patients have exceedingly poor 5 year survival rates indicating the need to develop novel chemotherapeutic drugs which could find their use either as sole therapy or in combination with already existing drugs.

The HIV (human immunodeficiency virus) infection is characterized by inherently increased risk of multiple blood and solid organ malignancies. Highly Active Anti-Retroviral Therapy (HAART) is the term used for intensive combination therapy used to treat patients with HIV infection. The combination typically consists of reverse transcriptase inhibitors (e.g. Zidovudine) and protease inhibitors (e.g. ritonavir, nelfinavir). Use of HAART has resulted in substantial reductions in progression of HIV to AIDS, reduction in opportunistic infections, hospitalizations, and deaths [3]. Interestingly, recent observations point to a decreasing incidence of neoplastic lesions in patients using HAART. [4-6] In the Swiss HIV Cohort Study Clifford et. al., [7] reported that in HAART users, the standardized incidence ratio for Kaposi Sarcoma decreased to 25.3 (95% CI = 10.8 to 50.1) as compared to 239 (95% CI = 211 to 270) in non HAART users. Many other investigators have subsequently reported similar associations of potential anti-neoplastic impact of HAART. [8-10] Even before the above mentioned studies were published, the anti-neoplastic properties of ritonavir (which is a protease inhibitor and forms an integral part of HAART), had already been demonstrated in some cancers. Specifically, Ritonavir induced apoptosis in tumor cell lines of lymphoblastoid origin, including lymphoma cells and myeloid leukemia cells, fibrosarcoma and mastocytoma cells as well as immortalized Kaposi's-sarcoma cell lines [11,12]. No effect on proliferation or survival was observed with non-tumor cells, including non-transformed immortalized fibroblasts or primary macrophages [13,14].

PI3K/AKT pathway is an important regulator of cellular proliferation and survival, and plays a central role in the progression and metastasis of various human cancers [15,16]. This pathway is activated in wide range of tumors but not in normal tissues. We hypothesize that the inhibition of this pathway with RNA*i *together will ritonavir treatment may offer better tumor regression. RNA*i *is an innate gene-silencing mechanism evolved to protect against viruses initiated by 19 bpl double-stranded RNA molecules (short interfering RNA) homologous to the sequence of the target gene that mediate post-transcriptional gene-silencing [17,18]. Introduction of chemically synthesized siRNAs can mimic gene silencing target genes. Loss of function studies can be done using siRNA technology for any given gene to assess the function of a gene.

The objective of the current study is to assess the anti-neoplastic impact of ritonavir, and to delineate the underlying mechanisms. Development of clinically approved HIV drug, ritonavir as an effective adjuvant therapy in ovarian cancer by drug repositioning could accelerate the process of traditional drug development in oncology.

## Results

### Ritonavir acts as antiproliferating agent for ovarian cancer cell lines

**Figure 1:**
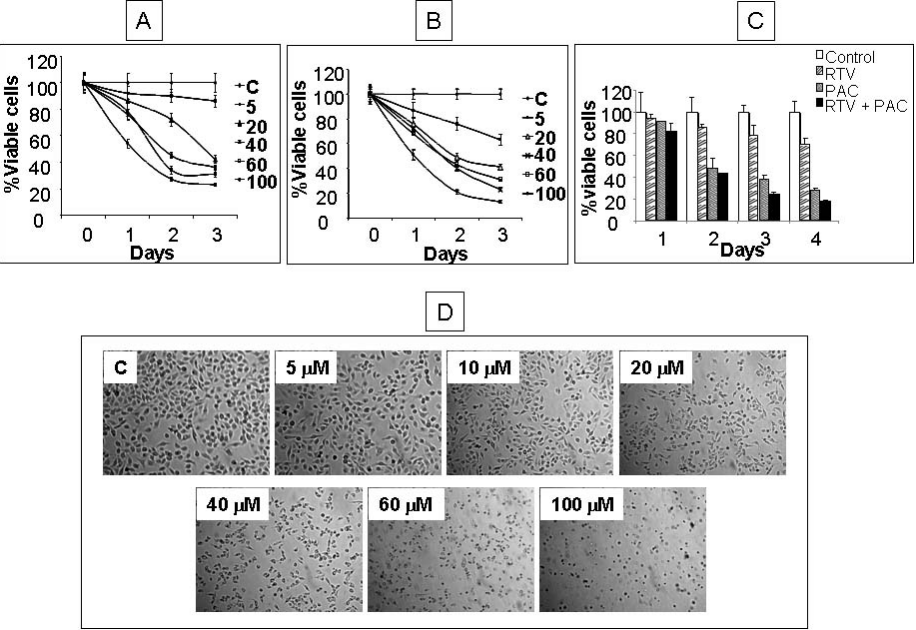
**Effect of ritonavir on the growth of ovarian cancer cell lines**. MDAH-2774 cells (A) and SKOV-3 cells (B) were cultured for indicated times at various concentrations of the ritonavir. Cell growth was assessed by CCK-8 cell proliferation assay method and is expressed as a percentage of control (DMSO treated cells) and represents the mean of triplicate cultures. C. MDAH-2774 cells were incubated with either 15 μM ritonavir (RTV) or 1 μM paclitaxel (PAC) or both and cultured for 4 days. Data is representation of three independent experiments. D. MDAH-2774 cells were incubated with indicated RTV concentrations for 48 hrs and were photographed under a phase contrast microscope. Representative fields of two independent experiments are shown.

We tested the cytotoxicity of the drug ranging from 5–100 μM on MDAH- 2774 and SKOV-3 ovarian cancer cell lines over 3 days exposure. A dose dependent inhibition of cell proliferation in both the cell lines was observed as shown (Fig. 1A &1B). Over a 3 day period with 20 μM ritonavir resulted in the cell death of over 60% for MDAH-2774 compared with 55% in case of SKOV-3 cell line. Further we tested if ritonavir can enhance the cytotoxicity of paclitaxel which is commonly used for the treatment of ovarian cancers. MDAH-2774 cells were tested with paclitaxel over 4 day period with and without ritonavir. Approximately 1 μM paclitaxel resulted in 40% cell death in 3 day period. We observed a time dependent decline in survival of MDAH-2774 cells in combination experiments with 10 μM ritonavir. With 1 μM paclitaxel and 10 μM ritonavir resulted in over 50% cell death in 2 day period in comparison with 20 μM ritonavir alone which takes 3 days to kill 50% of the cells (Fig. 1C). The data clearly indicates that ritonavir can significantly enhance effect of paclitaxel in ovarian cancer cells. Further the extent of cell death with ritonavir increased a function of time in all the tested doses ranging from 1 to 100 μM in 48 h as observed by phase contract microscopy in MDAH-2774 cell line (Fig. 1D).

### Ritonavir induces apoptosis MDAH-2774 cells

**Figure 2:**
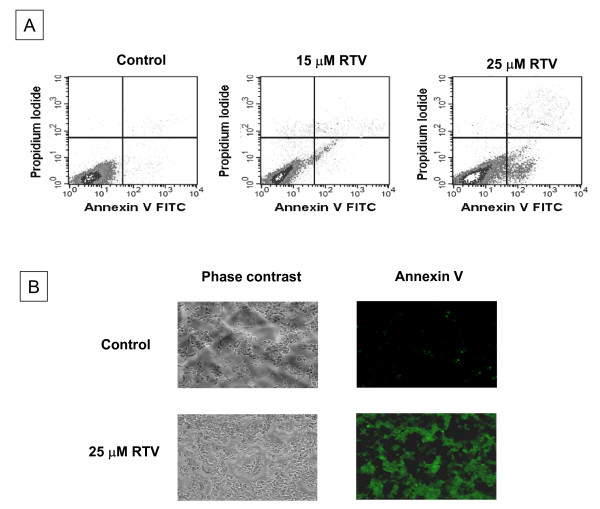
**Analysis of apoptotic cells**. Apoptotic cells were analyzed by using Annexin V FITC apoptosis ditection kit (Calbiochem, Gibbstown, NJ). A. Control and itonavir (15 and 25 μM) treated MDAH-2774 cells (1 × 10^5 ^cells) were fixed stained both with Annexin V FITC and with propidium iodide (20 μg/ml) were processed for flow cytometric analysis. A flow cytometer emitting an excitation light at 488 nm was used to quantify the Annexin V-FITC and propidium iodide signals. Log of FITC signal represented on X axis and log of propidium iodide fluorescence signals were plotted on Y axis. B. MDAH-2774 cells were mixed with annexin V-biotin, incubated for 15 min at room temperature, and treated with streptavidin conjugated to FITC. Apoptotic cells within the same microscopic field were viewed and photographed by phase contrast and by fluorescence emitted at 518 nm (FITC filter) were taken. Using the FITC filter, early apoptotic cells (positive for Annexin V-Biotin-FITC staining) appear bright green.

Annexin V is a Ca^2+ ^dependent protein with high affinity for membrane phospholipids [19] that has been used to identify apoptotic cells and has been validated in the literature. Control and treated cells were prepared for bivariate analysis using fluorescence-activated cell sorter system (FACS). We observed that the ratio of late apoptotic cells increased with the increased dose of ritonavir treatment: approximately 8% with 15 μM treatment and 20% with 25 μM treatment for 48 h as compared to early apoptosis, which increased from 9% to 36% (Fig. 2A). Further as analyzed by the fluorescent microscope after 48 h exposure to ritonavir, 80% of the cells stained positive for annexin V, whereas controls were less than 5% annexin V positive (Fig. 2B).

### Ritonavir enhances pro-apototic signals while inhibiting anti-apotitic bcl-2 expression

**Figure 3:**
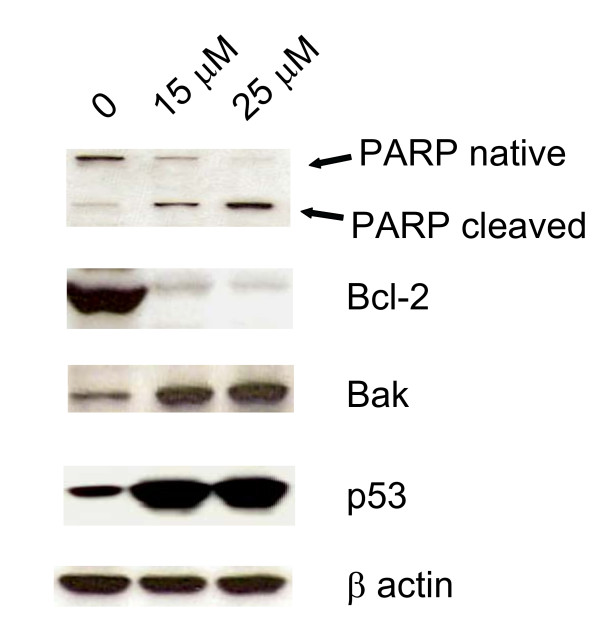
**Analysis apoptosis related proteins with the treatment of ritonavir**. MDAH-2774 cells treated with 15 μM ritonavir for 48 h were harvested and cell lysate was prepared. Approximately 10 μg of total cell extracts of control and ritonavir treated MDAH-2774 cells were resolved by SDS-PAGE, transferred to nitrocellulose membrane; and probed with PARP, Bcl-2, Bak and p53. β actin was used as a loading control. Native and cleaved PARP are indicated by arrows.

The balance between pro and anti-apoptotic signals decides the fate of the cells and therefore we examined the expression levels of genes that are involved in the apoptotic pathway by western blot analysis. Approximately 10 fold increase in the expression of p53 was observed by western blot analysis in the same samples (Fig. 3). Primary cellular function of Poly (ADP-ribose) polymerase-1 (PARP) is not entirely certain, never the less it plays an important role in apoptosis as a cellular response to chemotherapeutic and DNA damaging agents [20]. PARP cleavage is an indirect way of semi-quantitative measurement of the activated caspase-3 and a direct measurement of the extent of the apoptosis. Western blot analysis revealed dose dependent activation of the caspase-3 as determined by its cleavage of PARP. Treatment for 48 h resulted in a dose-dependent cleavage of the 117-kDa PARP to the smaller 85-kDa product. Cleavage was first seen at 15 μM of ritonavir, with further increases in the levels of the 85-kDa cleavage product seen with 25 μM. A dose-dependent increase in the expression of the pro-apoptotic protein Bak with concomitant inhibition of anti-apoptotic protein Bcl-2 was also observed (Fig 3).

### Ritonavir causes cell cycle arrest and blocks S phase entry of MDAH-2774 cells in cultures

**Figure 4:**
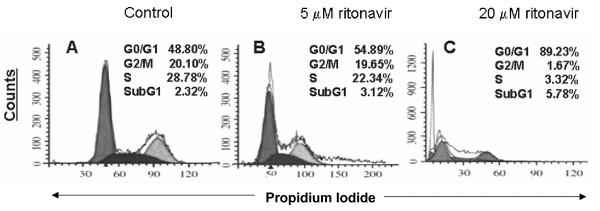
**Effect of ritonavir on DNA synthesis of MDAH-2774 cells**. MDAH-2774 and normal human fibroblasts were seeded at 10^6 ^cells and grown serum-free medium for 48 h for cell cycle synchronization. The cells were then treated with either vehicle DMSO (A) or 5 μM ritonavir (B) or 20 μM ritonavir (C). Cells were further incubated in presence of serum for 24 h followed by fixation, staining and cytometric analysis using Cell cycle phase determination kit (Cayman chemical company, Ann Arbor, MI).

Ritonavir induced G2/M arrest in a dose-dependent manner (Fig. 4A, B and 4C) in MDAH-2774 but not in normal human fibroblasts (data not shown). Remarkably, the proportion of cells in the G0/G1 phase arrest increased from 48.8% to 89.2% while S phase of cells decreased from 28.7% to 3.3% with the treatment of 20 μM ritonvir within 24 hrs (Fig. 4A, B and 4C). Further we observed dose dependent inhibition in the S phase cells indicative of the inhibition of DNA synthesis in MDAH-2774 cells in dose dependent manner.

### Ritonavir mediated perturbations in cell cycle regulatory proteins

**Figure 5:**
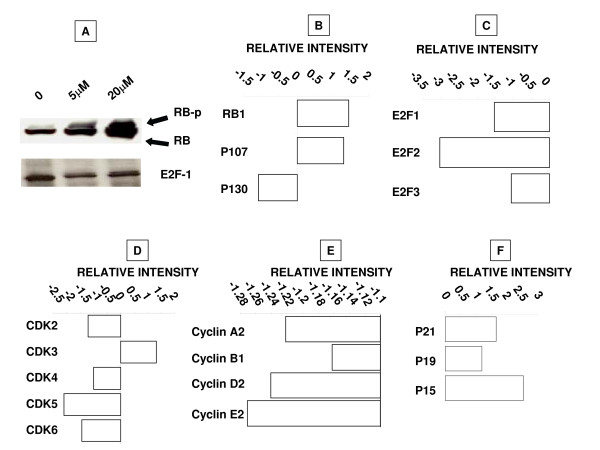
**Analysis of cell cycle regulatory proteins**. A. Approximately 10 μg of protein extracts of control and 5 μM and 20 μM ritonavir treated MDAH-2774 cells for 48 h were resolved by SDS-PAGE, transferred to nitrocellulose membrane; and probed with RB and E2F-1. β actin was used as a loading control. RB-p: hyper phosphorylated retinoblastoma protein; RB: Under phosphorylated retinoblastoma protein. MDAH-2774 cells treated with 15 μM ritonavir for 48 h were harvested and total RNA was isolated to generate cRNA and was hybridized to Whole Human Genome (G4112A) arrays (Agilent Technologies, Santa Clara, CA) according to the manufacturer's protocol. B. Analysis of RB family of pocket proteins. C. Analysis of E2F family of transcription factors. D, E and F represent the gene profile analysis of the expression of CDKs and Cyclins and CDKIs respectively that are involved in G0 to G1 phase transition.

Since we observed a significant increase in the population of G0/G1 phase of the cells with the treatment of ritonavir, we evaluated genes that control the cell cycle progression at G0/G1 phase. The cell cycle progression at G0/G1 phase is inhibited by active under-phosphorylated form retinoblastoma protein (RB) which sequesters growth promoting E2F-1 transcription factor. To evaluate if the observed block of S phase entry is due to the activation of RB protein, we first analyzed the levels of phosphorylation of RB and expression of E2F-1 by western blot analysis. We observed inhibition of phosphoryation status of the RB in response to ritonavir in dose dependent manner compared with control along with decrease in the E2F-1 protein levels (Fig. 5A). Gene expression analysis of RB and its related tumor suppressor proteins revealed an increase of 1.44 folds in the RB expression and 1.30 folds of p107 expression but there is decrease in the expression of p130 by 1.1 folds (Fig. 5B). Further we analyzed expression levels of three of the E2F family of proteins, E2F-1, 2 and 3 which interact with RB and as expected we observed 1.53 fold, 3.05 folds and 1.05 folds reduction in the expression levels of E2F-1, 2 and 3 respectively (Fig. 5C). Cyclins and cyclin dependent kinases (CDKs) exhibit distinct expression patterns which contribute to the temporal coordination of each event in cell cycle progression. G1 phase of cell cycle is regulated mainly by cyclin D, E and CDK2, 4 and 6. Ritonavir treatment resulted in the decreased expression levels of G1 phase cyclins and CDKs corroborating inactivation of RB proteins (Fig. 5D and 5E). Further we observed increased expression cyclin dependent kinase inhibitors (CDKIs) which bind and inhibit the activity of cyclin/Cdk complexes and negatively regulate cell cycle progression (Fig. 5F).

### Ritonavir inhibits AKT pathway leading to apoptosis in MDAH-2774 cells

**Figure 6:**
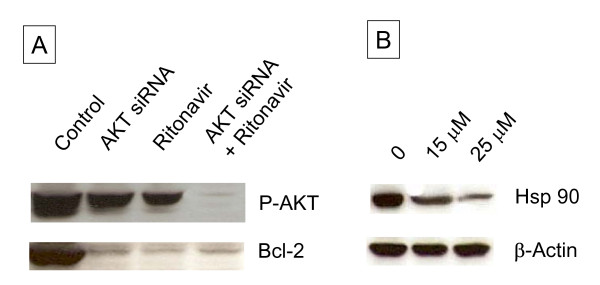
**Wester blot analysis of AKT siRNA treated MDAH-2774 cells**. A. Approximately 10 μg of protein extracts of control and treated samples of MDAH-2774 cells as indicated were resolved by SDS-PAGE, transferred to nitrocellulose membrane; and probed with Bcl-2. β actin was used as a loading control. B. SDS-PAGE analysis of ritonavir treated samples of MDAH-2774 cells.

AKT, plays a critical role in controlling the balance between survival and apoptosis and plays a significant role in insulin stimulation of glucose transport[21]. Earlier work with protease inhibitors showed inhibition of AKT in breast and hematological malignancies [22-24]. We tested if ritonavir inhibited AKT signaling in ovarian cancer cell lines using synthetic siRNA as known inhibitor for AKT. We found that ritonavir was more efficacious in decreasing phosphoryated AKT than siRNA. Significant reduction in the expression of AKT was also observed transfected with siRNA, compared with scrambled siRNA transfected control cells. Furthermore ritonavir has synergistic effect on reducing AKT expression when treated together AKT siRNA. In addition, the suppression of AKT both ritonavir treatment and AKT siRNA decreased the expression of anti-apoptotic Bcl-2 expression (Fig. 6A). Further we observed a dose dependent decrease in the Hsp90 levels with ritonavir treatment. Heat shock protein 90 (Hsp90) binds to AKT and protects it from being inactivated by protein phosphatase 2A-mediated dephosphorylation (Fig. 6B).

**Figure 7:**
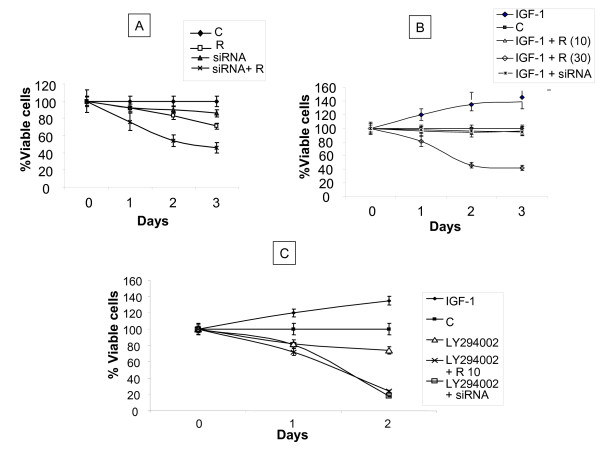
**Effect of AKT siRNA, ritonavir and modulators of AKT signaling on the growth of ovarian cancer cell lines**. Cells were cultured for indicated time points with various concentrations of the ritonavir. Cell growth was assessed by CCK-8 cell proliferation assay method and is expressed as a percentage of control (DMSO treated cells) and represents the mean of triplicate cultures. (R-ritonavir) A. Effect of ritonavir, siRNA and in combination; B. Effect of ritonavir and siRNA in presence of IGF-1. C. Effect of ritonavir in the presence of AKT inhibitor LY294002.

We next examined the effect of the inhibition of AKT expression on cell proliferation and apoptosis in ritonavir-treated MDAH-2774 cells. In order to investigate the possible additive effects of the inhibition of cell proliferation by AKT siRNA and ritonavir, we chose lower doses for the treatment. Ritonavir at 5 μM inhibited cell proliferation by approximately 20% but the cell death was dramatically increased to 60% when it was combined with 100 nM AKT siRNA. AKT siRNA transduction by itself inhibited the cell proliferation by approximately 15% (Fig. 7A). In order to further confirm our findings of involvement of AKT pathway with ritonavir treatment, serial treatments with IGF-1 (known AKT stimulator) and LY294002 (known inhibitor of AKT/PIK) pathway were carried out. When exposed to IGF-1, cell growth was increased by over 30%. Exposure of ritonavir down regulated the IGF-1 induced growth of the cells. We further observed that the AKT siRNA inhibition of IGF-1 induced cell growth was more pronounced than ritonavir (Fig. 7B). As expected, treatment with IGF-1 antagonized the effects of ritonavir and anti-AKT siRNA, whereas treatment with LY294002 potentiated the effects of ritonavir and anti-AKT siRNA (Fig. 7C).

### Ritonavir inhibits cell motility and invasiveness

**Figure 8:**
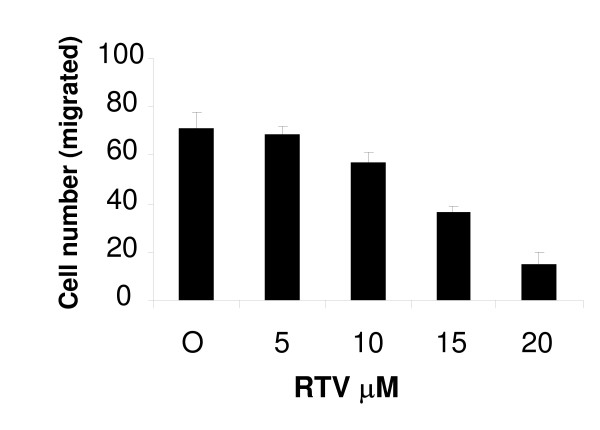
**Analysis of cell migration and invasion**. MDAH-2774 cells were cultured for 24 h, trypsinized and seeded in boyden chambers and treated with various concentrations of ritonavir as described in methods. Migrated cells through the membrame with different concentrations of ritonavir were stained and counted under microscope. Results of three independent experiments were plotted.

Cell migration and invasiveness are directly related to metastasis. We determined migration of MDAH-2774 cells in a modified Boyden chamber. Cells migrated through membranes covered with matrigel from upper chamber with various ritonavir concentrations to a lower chamber filled with medium only. We observed a progressive decrease in the cell migration through membrane with the ritonavir treatment from up to 20 μM. Treatment with 15 μM ritonavir decreased the cell invasion through the matrigel by 50% within 16 hours (Fig. 8). The decrease in the migration did not appear to be due to cytotoxicity since ritonavir (15 or 20 μM) showed no inhibitory effect on cell proliferation in 16 hr as determined by cytotoxic assay (data not shown).

## Discussion

Finding new indications for the already existing compounds, called drug repositioning that takes advantage of the existing data on pharmacokinetics, toxicity and dosage escalation studies in humans. Drug repositioning can potentially have tremendous cost savings and can expedite movement of a drug from bench to bedside in a relatively short amount of time [25]. For example lenalidomide, an analogue of thalidomide was originally marketed for morning sickness that is now 'repositioned' and approved for therapy of multiple myeloma [26]. This is a prime example of the immense potential of drug repositioning. Likewise oral hypoglycemic rosiglitazone [27], immunosuppressant drug rapamycin [28], and the birth control hormone medroxy-progesterone acetate [29] are also being tested for 'repositioning' to be used as anti-cancer agents. Ritonavir is an FDA approved drug for HIV treatment, being used well over a decade with tolerable side effects [30].

Ovarian cancer is the deadly form of gynecologic malignancy with exceedingly poor 5 year survival rates [2] and is the subject of intense research for development of newer antineoplastic compounds which could be used either as a sole or adjuvant therapy. Further, newer compounds may hold even greater promise in drug resistant and relapsing ovarian cancer where the efficacy of the existing chemotherapeutic agents is marginal, at best.

Here for the first time, we show that ritonavir acts as an effective anti-proliferating agent for the ovarian cancers cells *in vitro *by inducing growth arrest and apoptosis providing insights into molecular mechanisms. Further, it also exhibits the potential to inhibit invasion and migration of these cell lines. Although paclitaxel and carboplatin have good response rates, there are limited treatment options in case of disease relapse where majority of patients become refractory to conventional chemotherapy due to the generation of drug resistant phenotype [31]. In addition we document an additive effect of cell killing when ritonavir combined with paclitaxel.

Retinoblastoma protein (RB) is an important tumor suppressor protein that control progression through the late G1 phase of the cell cycle and, thereby, the commitment to enter the S phase[32,33] Also, E2F-1 transcription factor that is necessary t o drive the cell into S phase.[32,34,35] Cyclins and cyclin-dependent kinases (CDKs) regulate the activity of RB by phosphorylation that controls the progression through G1 [34-36]. Since we observed elevated levels of under-phosphorylated RB, we speculated the lower levels of CDK-2, 4 and 6, one of the important proteins responsible driving cell cycle progressions through G0/G1 phase of cell cycle by phosphorylation of RB. As expected, gene profile analysis of ritonavir treated cells showed down modulation of CDKs and cyclins that are gate keepers of G0/G1 phase of cell cycle. The p21WAF-1/Cip1 inhibits CDK-4, thus preventing both phosphorylation of RB and the release of transcriptionally active E2F-1. Our observations of lower levels of CDK inhibitors and inhibition of RB phosphorylation along with increased levels of expression of E2F-1 transcription factor in response to ritonavir corroborate the results of cell cycle analysis where cells entering S-phase were reduced by over 25%.

Numerous studies show that the PI3K/AKT pathway is constitutively over-expressed in ovarian cancers, apart from several other common human cancers [37]. Activation of AKT is a key event in producing chemo-resistant phenotype through a variety of pathways [38], whereas inhibition of AKT sensitizes chemo-resistant cells to cisplatin induced apoptosis. Our data corroborates with the observations that increased levels of AKT contributes to chemo-resistance by attenuating p53-mediated PUMA up-regulation and phosphorylation of p53, which are essential for sensitivity to cisplatin-induced apoptosis [39]. Our data indicated that ritonavir inhibits phosphorylation of AKT followed by effective apoptosis. Taken together, these findings suggest that ritonavir may have useful role as an anti-AKT agent in treatment of ovarian cancer in general, but more specifically in relapsed patients due to drug resistance phenotype generation which is attributed to AKT. Further, the Bcl-2 inhibition appeared to be mediated through an AKT dependent pathway, as treatment of anti-AKT siRNA had similar results as ritonavir in Bcl-2 down regulation.

One of the important reasons for high mortality in ovarian cancer patients is the late stage at which the disease is diagnosed, namely FIGO III [40], where tumor cells would have already traversed the peritoneal cavity by migration and invasion. Effect of ritonavir in inhibiting the invasion and migration of the ovarian cancer cell lines adds another dimension in its anticancer properties and may be especially useful in ovarian cancer as trans-peritoneal spread is a key event in this cancer. We are currently studying the precise molecular pathways involved in migration inhibition seen with ritonavir in *in-vivo *models.

Ritonavir has been in use for over a decade in the treatment of HIV patients with acceptable toxicity profiles, the primary impetus for this work has been to evaluate the re-positioning of the drug to ovarian cancer chemotherapy. An important aspect of repositioning of ritonavir from HIV therapy to cancer therapy will be the achievable dose in cancer patients. Ritonavir blood plasma levels in HIV patients normally observed at 15 μM [41] and much higher concentrations of over 45 μM were also observed in individual patients [41,42]. We observed the growth inhibitory effects of ritonavir in ovarian cancer patients in the range of 5 to 20 μM which is lower than the plasma concentrations observed in HIV patients.

Studies to further elucidate mechanism, specifically cell signaling target modulation, are ongoing in our lab. The determination of synergistic or additive effects in conjunction with conventional chemotherapeutic regimens represents a putative application for ritonavir at its so far known non-toxic concentrations. This would accelerate the process of drug development for a disease that has highest mortality rate among gynecological cancers.

## Conclusion

Ovarian cancer poses many treatment difficulties as it is often undetectable in its early stages, and therefore diagnosis usually occurs when surgical treatment is no longer an effective option emphasizing the need for novel, non-toxic and effective treatments. Here we present the evidence that ritonavir; an FDA approved drug for human use for HIV effectively induces cell cycle arrest and apoptosis by inhibiting AKT pathway and retinoblastoma phosphorylation. Further we observed an additive effect of ritonavir in killing ovarian cancer cells when used in conjunction with paclitaxel showing its potentials to be 'repositioned' for ovarian cancer as an adjuvant therapy.

## Materials and methods

### Reagents and antibodies

Ritonavir was obtained from Sequoia Research Products Limited (Pangbourne, UK) and dissolved in dimethyl sulfoxide (DMSO). Stock solutions were freshly prepared in DMSO (0.001%) and added to the cell cultures to obtain the indicated final concentrations. DMSO alone (0.001%) was found to have no significant effect on cellular function. Following antibodies were used: Retinoblastoma & E2F-1 antibodies from Millipore (Danvers, MA); Cyclins, CDKs, poly (ADP-ribose) polymerase (PARP) and actin antibodies from Santa Cruz Biotechnology, (Santa Cruz, CA). Antibodies against phospho-AKT, caspases, Insulin like growth factor 1 (IGF-1) were obtained from Cell Signaling Technology (Beverly, MA). SignalSilence AKT siRNA inhibition kits were purchased from Cell Signaling Technology (Beverly, MA).

### Cell lines and culture

Ovarian cancer cell lines, MDAH-2774 and SKOV-3 (American Type Culture Collection, Manassas, VA) were propagated in McCoy's 5A medium and normal human fibroblasts were propagated in DMEM medium, both were supplemented with 10% fetal bovine serum, 2 mM L-glutamine, 100 units/ml penicillin, and 100 μg/ml streptomycin (ThermoFisher Scientific, Pittsburgh, PA). Cells were cultured in a humidified atmosphere with 5% CO2 at 37°C. Trypsin (0.25%)/EDTA solution was used to detach the cells from the culture flask for passing the cells.

### Cell proliferation assays

Standard prototype growth curves and number of viable cells were determined for each cell line (treated and control groups) in triplicate experiments by the Cell Counting Kit-8 (CCK-8) (Dojindo, Gaithersburg, MD) according to manufactures' instructions. Briefly, cells were plated at a density of 3,000 per well in 96-well plates in a total of 100 μl medium and allowed to grow for 24 h. Ritonavir dissolved in DMSO was added, and the cells were allowed to grow for the indicated time. Growth of the cells in each set of a group was terminated by adding 10 ml of CCK-8 reagent, incubated for an hour and absorbance was read at 450 nm in a plate reader (FluoStar Optima, BMG Labtech). Growth curves were plotted as a percentage of the value of DMSO-treated controls minus the value of untreated cells on day 0. Day 2 & 3 values were considered for the determination of the 50% cell proliferation inhibition (IC50) for a given treatment. In some cases parallel manual count was also performed with trypan blue and counting by exclusion method using a Hemocytometer. The findings confirmed CCK-8 assay results. Human fibroblasts were similarly treated as cancer cells to display differential cytotoxicity at any given dose.

### Analysis of apoptotic cells

Apoptotic cells were analyzed by using Annexin V FITC apoptosis detection kit (Calbiochem, Gibbstown, NJ). Ritonavir treated MDAH-2774 cells (1 × 10^5 ^cells) were trypsinized, washed with cold PBS, fixed with 70% ethanol, and stored at -20°C until use. The fixed cells were stained with propidium iodide (20 μg/ml) with RNaseA (10 μg/ml) and incubated at room temperature for 30 min in the dark. The DNA content of the cells was analyzed by flow cytometer using the fluorescence-activated cell sorter system (FACS) and sub G1 population was considered to represent apoptotic cells. Fluorescence microscope (Zeiss, AXio CamMRm Observer. A1) was used for visual analysis of apoptotic cells. Propidium iodide (PI) was added to discriminate early apoptotic cells from late apoptotic or necrotic cells. For the fluorescent microscopy, after incubating the cells with Ritonavir at the indicated dose concentrations for 48 hours, the cells were trypsinized and washed twice with cold PBS (0.15 mol/L, pH 7.2). Centrifugation was performed at 5000 c/min for 5 min, and the pellet was resuspended in 1 × binding buffer at a density of 1.0 × 10^5 ^–1.0 × l0^6 ^cells per mL. Further incubation was performed with 5 μL of FITC-conjugated annexin V and 5 μL of PI for 15 min in the dark. 400 μL of 1 × binding buffer was added to each sample tube, and the samples were analyzed by FACS.

### Cell cycle phase determination

MDAH-2774 cells were seeded at 1 × 10^6 ^cells in 10 cm dishes and the culture medium changed to serum-free medium for 24 h to facilitate cell cycle synchronization. Cell cycle analysis was conducted using Cell cycle phase determination kit (Cayman chemical company, Ann Arbor, MI). Cells were treated with 5 or 20 μM ritonavir and further incubated in medium containing 10% serum. Later cells were trypsinized, washed with PBS and re-suspended in 1 ml of assay buffer and 1 ml of fixative both provided with the kit. After 2 h incubation, cells were centrifuged at 500c for 5 min and cell pellet was retained. Pellet was suspended in 0.5 ml of staining solution that contained PI and incubated for 30 min at room temperature in the dark. Samples were analyzed in FL2 channel of flow cytometer with a 488 nm excitation laser.

### Western blotting

With 4–15% SDS Tris-Glycine gels, western transfer of proteins to nitrocellulose papers was conducted with iBlot dry blotting device (InVitrogen Corp, Carlsbad, CA). Blocking agent was 3% non fat milk powder and the secondary antibodies conjugated to HRPO (Santa Cruz Biotechnology, Santa Cruz, CA) were used. Enhanced chemiluminescence detection kit from Amersham Pharmacia, Uppsala, Sweden was used.

### Transfection of Akt small interfering RNA (siRNA)

SignalSilence AKT siRNA inhibition kit (Cell Signaling Technology Beverly, MA) that specifically inhibits the expression of both AKT 1 and AKT 2, was utilised to downregulate AKT protein in MDAH-2774. Briefly, MDAH-2774 cells were transfected with 100 nM siRNA with the transfection reagent provided in the kit. Cells were harvested after 48 hrs and analyzed for the expression of AKT, Bcl-2 and actin antibodies. Controls were transfected with non-specific SiRNA and grown under similar conditions. Similar to SiRNA, inhibition of AKT signaling was achieved by LY294002 whereas forced induction of the Akt pathway was achieved by IGF-1 treatment. The treatment with AKT SiRNA, IGF-1 and LY294002 was conducted to investigate the AKT/PIK pathway involvement in Ritonavir treatment of cell cultures.

### In vitro migration and wound-healing Assays

Cell migration was determined with a modified Boyden chamber assay with filters of 8-μm pore size were used. Briefly, MDAH-2774 cells (10^5^/500 μL) were added into the upper compartment of a migration chamber with various concentrations of ritonavir. The chamber was then incubated at 37°C in a 5% CO_2_. After 18 h, the non-migrated cells on the upper surface of the membrane were removed using cotton buds. The underside of the chamber was washed twice with in PBS, fixed by 4% formaldehyde for 20 min and stained by DIPA or crystal violet (0.1% w/v) for 15 min. The number of migrated cells at other side of the compartment was counted under a microscope for nine random fields. The assays were performed in triplicate. For wound healing assays, MDAH-2774 cells were plated at equal density in and grown to 80% confluency. Using a sterile pipette tip, wounds were generated. Cells were then rinsed with medium and replaced with the fresh medium. Areas of wound were marked and photographed at various time points with a phase-contrast microscope.

### Gene Expression Profiling

MDAH-2774 cells treated with 15 μM ritonavir for 24 h, were harvested and total RNA was isolated utilizing an RNeasy kit (Qiagen Inc., Valencia, CA), reverse-transcribed to get cDNA using the "Superscript II RT kit" (Invitrogen™ Life Technologies, Carlsbad, CA). cDNA was used in an *in vitro *transcription reaction to synthesize cRNA utilizing "ENZO RNA labeling kit (Enzo Diagnostics, Inc., Farmingdale, NY). Labeled cRNA was purified with the RNeasy Mini-kit (Qiagen Inc., Valencia, CA) and quantitated. Purified cRNA (15 μg) was hybridized to Whole Human Genome (G4112A) arrays (Agilent Technologies, Santa Clara, CA) according to the manufacturer's protocol. The G4112A set consists of arrays representing approximately 44,000 human genes. Total RNA was amplified using Agilent Low Input Linear Amplification Kit according to the process outlined by the manufacturer (Agilent Technologies, Santa Clara, CA). Amplified target cRNA (1–5 μg) was labeled with either cyanine-5 or cyanine-3 using ULS RNA Flurorescent Labeling Kit according to the manufacturer's protocol (Kreatech Biotechnology, Amsterdam, The Netherlands). Concentration of labeled cRNA and the label incorporation was determined by Nanodrop-1000 spectrophotometer. Labeled cRNA was fragmented and hybridized overnight to Agilent whole Human Genome arrays (4 × 44 K format) according to Agilent protocol. The arrays were scanned using Agilent scanner (G2505B) and data was extracted using Agilent's Feature Extraction Software.

## Authors' contributions

SK and CSB initiated the project, generated growth curves and conducted AKT related experiments. SC and AQ conducted cell migration assays and wound healing assays. MAS and CSS helped with mRNA isolation and gene profile analysis. JP and SS conducted western blot analysis and immunoprecipitations. RM and JMM participated in the design of the study and co-ordinated the experiments. MP and DWW, RBB conceived of the study, and participated in its design and coordination and helped to draft the manuscript. All authors read and approved the final manuscript.
